# Epigenetic Markers Are Associated With Differences in Isocyanate Biomarker Levels in Exposed Spray-Painters

**DOI:** 10.3389/fgene.2021.700636

**Published:** 2021-07-14

**Authors:** Laura W. Taylor, John E. French, Zachary G. Robbins, Leena A. Nylander-French

**Affiliations:** ^1^Department of Environmental Sciences and Engineering, Gillings School of Global Public Health, University of North Carolina at Chapel Hill, Chapel Hill, NC, United States; ^2^Nutrition Research Institute, University of North Carolina at Chapel Hill, Chapel Hill, NC, United States

**Keywords:** epigenome-wide association study (EWAS), epigenetics (DNA methylation), biomarkers, exposure assesment, isocyanates, bioinformatics, gene–environment interactions

## Abstract

Isocyanates are respiratory and skin sensitizers that are one of the main causes of occupational asthma globally. Genetic and epigenetic markers are associated with isocyanate-induced asthma and, before asthma develops, we have shown that genetic polymorphisms are associated with variation in plasma and urine biomarker levels in exposed workers. Inter-individual epigenetic variance may also have a significant role in the observed biomarker variability following isocyanate exposure. Therefore, we determined the percent methylation for CpG islands from DNA extracted from mononuclear blood cells of 24 male spray-painters exposed to 1,6-hexamethylene diisocyanate (HDI) monomer and HDI isocyanurate. Spray-painters’ personal inhalation and skin exposure to these compounds and the respective biomarker levels of 1,6-diaminohexane (HDA) and trisaminohexyl isocyanurate (TAHI) in their plasma and urine were measured during three repeated industrial hygiene monitoring visits. We controlled for inhalation exposure, skin exposure, age, smoking status, and ethnicity as covariates and performed an epigenome-wide association study (EWAS) using likelihood-ratio statistical modeling. We identified 38 CpG markers associated with differences in isocyanate biomarker levels (Bonferroni < 0.05). Annotations for these markers included 18 genes: ALG1, ANKRD11, C16orf89, CHD7, COL27A, FUZ, FZD9, HMGN1, KRT6A, LEPR, MAPK10, MED25, NOSIP, PKD1, SNX19, UNC13A, UROS, and ZFHX3. We explored the functions of the genes that have been published in the literature and used GeneMANIA to investigate gene ontologies and predicted protein-interaction networks. The protein functions of the predicted networks include keratinocyte migration, cell–cell adhesions, calcium transport, neurotransmitter release, nitric oxide production, and apoptosis regulation. Many of the protein pathway functions overlap with previous findings on genetic markers associated with variability both in isocyanate biomarker levels and asthma susceptibility, which suggests there are overlapping protein pathways that contribute to both isocyanate toxicokinetics and toxicodynamics. These predicted protein networks can inform future research on the mechanism of allergic airway sensitization by isocyanates and aid in the development of mitigation strategies to better protect worker health.

## Introduction

Isocyanate exposures are an occupational hazard of global concern and are associated with numerous acute and chronic health effects, principally isocyanate-induced asthma (Liu and Wisnewski, 2003; Malo and Chan-Yeung, 2009; Dao and Bernstein, 2018). Exposures can occur during the manufacture of or end-user application of polyurethane-based products including paints and coatings, spray-foam insulation, flexible and rigid foams, and furniture adhesives (Randall and Lee, 2002). Monomers and oligomers of toluene diisocyanate (TDI) and methylene diphenyl diisocyanate (MDI) are the most widely produced isocyanates (Randall and Lee, 2002). Classified as aromatic isocyanates, TDI and MDI are fast curing compounds primarily used for indoor polyurethane applications because they are susceptible to degradation by ultraviolet (UV) radiation (Bello et al., 2004). TDI and MDI monomer exposures and their associated health effects are well-studied due to their widespread use. Conversely, aliphatic isocyanates are highly resistant to UV radiation and thus, are preferentially used in exterior coatings. The most common aliphatic isocyanate, 1,6-hexamethylene diisocyanate (HDI) monomer and its oligomers, is a primary component of automotive clearcoat paints (Randall and Lee, 2002; Bello et al., 2004), posing an occupational exposure concern for spray-painters. The Health and Safety Executive from the United Kingdom reported that automotive spray-painters are 80 times more likely to develop occupational asthma than workers in other occupations, and there is no cure once a worker has become sensitized (HSE, 2014). Currently, there is limited research on exposures to HDI monomer and its oligomers, so we have focused our efforts on helping to fill this knowledge gap. Although monomers and oligomers of TDI, MDI, and HDI all contain two or more reactive isocyanate groups (–N = C = O), the reactivity of the isocyanate group differs in these chemicals due to differences in their size and structure (Randall and Lee, 2002; Bello et al., 2004). Only a subset of exposed workers will develop isocyanate-induced asthma though, which suggests that some workers’ genetics and/or epigenetics may lend them added protection against isocyanate sensitization.

Other researchers have investigated this and found significant genetic and epigenetic markers that are correlated with differences in the prevalence of TDI-, MDI-, and HDI-induced asthma in exposed workers. Genetics studies primarily indicate markers for genes involved in immune system response and metabolism pathways that impact workers’ prevalence of isocyanate-asthma, including *HLA* serotypes, *GST* genes, *NAT* genes, and *TGF-*α (Bignon et al., 1994; Balboni et al., 1996; Mapp et al., 2000, 2002; Piirilä et al., 2001; Wikman et al., 2002; Beghé et al., 2004; Kim et al., 2006; Choi et al., 2009; Yucesoy et al., 2012, 2014, 2015, 2016). Additionally, an epigenetic study by Ouyang et al. (2013) showed that there were differences in methylation levels in the promoter regions for two genes involved in inflammatory response, interferon-γ (*IFN-*γ) and interleukin-4 (*IL-4*), in workers with isocyanate-asthma when they were compared to exposed workers without isocyanate-asthma or to non-smoking exposed workers.

However, more research is needed on the processes that occur after isocyanate exposure and before asthma development. It is known that isocyanates react readily with water and amines, and the research on protein adducts and asthma risk indicates that the gene products GST, NAT, and/or CYP450 may be involved in the biotransformation and metabolism of isocyanates (Flack et al., 2010a), but little else is known. As such, an international conference in Maryland identified that more research is needed to “develop approaches to better understand the connections between isocyanate uptake, metabolism, immunogenicity and asthma pathogenesis” and to “assess the utility of new models for predicting human responses to exposure and risk assessment” (Lockey et al., 2015). Learning more about the protein pathways involved in the absorption, distribution, metabolism, and elimination (ADME) of isocyanates by evaluating inter-individual biomarker level variance before asthma pathology develops can help fill these persistent knowledge gaps.

Previously, we reported on a pilot study in which the association between epigenetic DNA methylation levels and the variability in isocyanate biomarker levels measured in occupationally exposed workers was investigated (Nylander-French et al., 2014), but to our knowledge, no other research has been done on this topic. Despite not finding other research on associations between epigenetics and isocyanate biomarker levels, there have been genetic studies showing an association with biomarker variability in workers. Genetic studies on TDI have shown associations between *GSTM1* and *P2RX7* genetic polymorphisms and TDI biomarker levels in exposed workers (Broberg et al., 2010; Broström et al., 2018). Additionally, our previous genome-wide association study (GWAS) on the association between genetics and exposure biomarker levels demonstrated that transcription regulation, calcium ion transport, vascular morphogenesis, and inflammatory pathways including the *TGF-*β pathway may be involved in the ADME of isocyanates after exposure and before isocyanate-asthma development (Taylor et al., 2020). For this epigenetic study, we expanded our 2014 pilot research (Nylander-French et al., 2014) by measuring the methylation of more CpGs and in more workers than before, while still using part of the cohort of workers included in our GWAS. In this study, we investigated associations between epigenome-wide methylation levels and isocyanate biomarker levels with the goal to enhance knowledge about which protein pathways impact isocyanate toxicokinetics.

## Materials and Methods

### Study Participants

This study included 24 workers who were exposed to 1,6-hexamethylene diisocyanate (HDI) monomer and HDI isocyanurate in polyurethane spray-paints in autobody repair shops in North Carolina and Washington. These workers are a subset of a well-defined cohort that has been described previously (Fent et al., 2009a,b; Flack et al., 2010b; Gaines et al., 2010a) and the correlation between genetics and biomarker levels in these workers has also been reported (Taylor et al., 2020). The 24 workers included in this study are described in Table 1. All were males aged 40 ± 10 years (range: 21–59 years) who had been painting for 20 ± 10 years (range: 0.5–35 years). Body surface area (BSA) was approximated using the approach by Haycock et al. (1978). Eighteen painters were White, two were Hispanic, one was African-American, one was Native American, one was Pacific Islander, and one was mixed European Indian American. Six of the workers self-reported atopy, including one worker reporting asthma and seasonal allergies, one reporting asthma and year-around allergies, three reporting only seasonal allergies, and one reporting skin allergies. One of the two workers who reported having asthma was currently taking asthma medication. Eight of the painters were smokers, seven were ex-smokers, and nine were never smokers. Informed consent from all workers was received prior to exposure surveys. The institutional review boards from the University of North Carolina at Chapel Hill Office of Human Research Ethics (Study #12-1195) and from Washington University Department of Social and Health Services (Study #A-013106) approved this study.

**TABLE 1:** Demographics of the spray-painter population.

Worker characteristics						
	**Age (years)**	**Weight (kg)**	**Height (cm)**	**BMI**	**BSA (m^2^)**	**Painting experience (years)**

Average ± STD (min – max)	40 ± 10 (21–59)	90 ± 16 (57–120)	177 ± 9 (157–196)	29 ± 4 (21–39)	2.1 ± 0.2 (1.6–2.4)	20 ± 10 (0.5–35)

**Ethnicity**						

	**White**	**Hispanic**	**African-American**	**Native American**	**Pacific Islander**	**European Indian American**

No. of workers	18	2	1	1	1	1

**Allergies and asthma history**						

	**Skin allergies**	**Seasonal allergies**	**Year-round allergies**	**Asthma**	**Chronic asthma**	**Asthma medication used**

No. of workers	1	4	1	2	0	1

**Smoking history**						

	**Current**		**Previous**		**Never**	

No. of workers	8		7		9	

*BMI, body-mass index; BSA, body surface area; STD, standard deviation; min, minimum; max, maximum.*

### Exposure Measurements

The only isocyanates that the workers were occupationally exposed to were HDI and its oligomers. Sampling and quantification methods for inhalation and skin exposures to HDI monomer and HDI isocyanurate and their exposure biomarkers have been published previously (Fent et al., 2009a,b; Flack et al., 2010b, 2011; Gaines et al., 2010a; Robbins et al., 2018), including the methods for this particular cohort (Taylor et al., 2020). Briefly, exposure levels and biomarker levels were measured for consenting spray-painters during spray-painting tasks conducted during a workday, with up to three workday visits total. The visits were spaced at least 3 weeks apart during the span of a year and were performed in order to increase statistical power by having multiple independent measurements for each worker (Rappaport et al., 1995; Lyles et al., 1997; Serdar et al., 2006).

Personal breathing-zone sampling and skin tape-strip sampling were used to quantify HDI monomer and HDI isocyanurate inhalation and skin exposures, respectively, during each paint task (Fent et al., 2009a,b). Air samples from the workers’ breathing-zone were captured using filter cassettes and the levels of HDI monomer and HDI isocyanurate were analyzed using high-performance liquid chromatography-mass spectrometry (HPLC-MS) (Fent et al., 2009a). Time-weighted average personal breathing-zone concentration (TWA; μg/m^3^) was adjusted with the Occupational Safety and Health Administration (OSHA) assigned protection factor (APF) based on the type of respirator worn by the worker during the painting task (Occupational Safety and Health Administration, 2009). The TWA was then multiplied by the time spent painting (minutes) and the average male breathing rate (0.0232 m^3^/minute) to estimate the daily inhalation exposure (μg) for each visit (Adams, 1993). Skin exposure to HDI monomer and to HDI isocyanurate was evaluated by collecting tape-strip samples, as described previously (Fent et al., 2009b). Tape-strip samples were taken on the dorsal side of each hand and on the dorsal and volar side of each lower arm for all workers. Samples from the neck were also collected when workers were not wearing hoods and samples from the wrists were collected when the worker’s wrists were left exposed during painting. HPLC-MS was then used to quantify the amount of each isocyanate on the tape-strip samples. Skin exposure was estimated as a mass of exposure (μg) by calculating the sum of HDI monomer or HDI isocyanurate in the tape-strips collected from the sampled body parts.

### Biomarker Measurements

The biomarkers measured were 1,6-diaminohexane (HDA) and trisaminohexyl isocyanurate (TAHI), which are the hydrolyzed products of HDI monomer and HDI isocyanurate, respectively. For plasma biomarker level evaluation, one blood sample was drawn near the end of each visit and was quantified using gas-chromatography mass-spectrometry (GC-MS) for HDA (Flack et al., 2010b) and nanoflow ultra-performance liquid chromatography coupled with nano-electrospray ionization tandem mass spectrometry (nano-UPLC-ESI-MS/MS) for TAHI (Robbins, 2019). The plasma level (μg) was calculated by multiplying the plasma biomarker concentration (μg/L) by the worker’s estimated plasma volume (L), which was determined using a male-specific factor of 0.04 l of plasma per kilogram of body weight (Vengelen-Tyler, 1996). For urine biomarker level evaluation, every urine void was collected during the full workday for each visit. Isocyanate biomarker levels were quantified for each urine sample using GC-MS for HDA (Gaines et al., 2010a) and nano-UPLC-ESI-MS/MS for TAHI (Robbins et al., 2018; Robbins, 2019). Biomarker levels in each urine sample was adjusted with creatinine level (μg/g creatinine) to account for differences in the workers‘ hydration levels (Gaines et al., 2010b).

To help satisfy normality assumption for statistical analyses, exposure and biomarker levels below the method detection limit (MDL) or limit of detection (LOD) were imputed to non-zero values using the equation (MDL/√2)/100 or (LOD/√2)/100, respectively. The geometric means of daily inhalation exposure, skin exposure, and plasma biomarker levels were calculated across the multiple visits for each worker for initial statistical tests in R. For urine biomarker levels, the geometric mean across visits of the average creatinine-adjusted concentration of all voids during each workday was calculated for each worker.

### Illumina MethylationEPIC BeadChip Array

Peripheral blood mononuclear cells (PBMCs) were collected for measuring DNA methylation level because they have a long survival time and they are exposed to circulating isocyanates absorbed through both the skin and inhalation routes, both of which are important pathways of exposure for spray-painters. Peripheral whole blood samples were collected from all workers using tripotassium ethylenediaminetetraacetic acid (K_3_EDTA) anticoagulant tubes. The PBMCs were separated from the blood sample using Ficoll^TM^ separation. QiaAmp Blood mini kits (Qiagen, Germantown, MD, United States) were then used to isolate DNA from the PBMCs. DNA quality and quantity was a minimum of 1 μg of genomic DNA at a concentration of 20 ng/μL in 60 μL of nuclease-free water. The DNA concentration was verified by PicoGreen or equivalent method. DNA samples from three workers were amplified using polymerase chain reaction (PCR). Bisulfite conversion was conducted using 500 ng of genomic DNA according to the manufacturer’s protocol for the Zymo EZ DNA Methylation kit (Zymo Research Corp., Irvine, CA, United States). DNA was added to Zymo MDilution buffer and incubated for 15 min at 37°C. CT-conversion reagent was then added and the mixture was denatured by heating to 95°C for 30 s followed by incubation for 1 h at 50°C. This denature/incubation cycle was repeated for a total of 16 h. After bisulfite conversion, the DNA was bound to a Zymo spin column and desulfonated on the column using desulfonation reagent according to the manufacturer’s protocol. The bisulfite-converted DNA was eluted from the column in 10 mL of elution buffer. The Center for Genomics and Personalized Medicine Research from Wake Forest University quantified the PBMC CpG methylation levels using the Infinium MethylationEPIC Kit (EPIC array) from Illumina (kit is available from: https://www.illumina.com/products/by-type/microarray-kits/infinium-methylation-epic.html). The EPIC array evaluates the methylation levels for 863,904 CpG loci, along with 2,932 non-CpG loci, and 59 SNP sites (Zhou et al., 2017), and it has been independently validated (Moran et al., 2016).

### Preprocessing of Epigenetic Data

The EPIC array idat files were uploaded into R version 4.0.1^1^ and preprocessed using the “mpreprocess” command in the ENmix package (Xu et al., 2016, 2020). This command performs Exponential-Normal mixture signal intensity background correction (ENmix) for background correction, REgression on Logarithm of Internal Control (RELIC) probes for dye bias correction, quantile normalization for normalizing methylated and unmethylated fluorescent intensities separately, and Regression on Correlated Probes (RCP) for probe-type bias adjustment (Pidsley et al., 2013; Niu et al., 2016; Xu et al., 2016, 2017). This approach mitigates background noise and systematic experimental bias (Xu et al., 2020). For quality control (QC), low quality data points, defined as having fewer than three beads or a detection *p*-value > 1E-6, were excluded. Additionally, 4,873 methylation sites with more than 5% low quality data points were removed. All 24 workers’ samples were verified to have <5% low quality CpG and all had bisulfite intensity >5,626.597. There were 861,532 methylation sites remaining after QC.

### Epigenome-Wide Association Study

We performed an epigenome-wide association study (EWAS) to determine the correlation between methylation levels and isocyanate-exposure biomarker levels for HDI monomer and HDI isocyanurate. Likelihood ratio (LR) tests were performed using the “lrtest” command within the lmtest package^2^. The LR test evaluates whether adding a variable to a linear regression model improves the fit of that model (Kohler, 1982). Inhalation and skin exposure were controlled for as covariates and the models were run both with and without age, smoking, and ethnicity as additional covariates. The LR tests evaluated the CpG methylation improved the fit of model 1 compared to model 2:

(1) Biomarker Level = Inhalation Exposure + Skin Exposure + Age + Smoking + Ethnicity + CpG Methylation
(2) Biomarker Level = Inhalation Exposure + Skin Exposure + Age + Smoking + Ethnicity

For smoking, non-smokers were coded as 0 and current smokers were coded as 1. For ethnicity, non-Latino whites were coded as 0 and other ethnicities were coded as 1. Further stratification of the ethnicity variable was not performed due to the small sample size. *p*-values were adjusted for multiple comparisons using the Bonferroni correction method (“p.adjust” command in R) in which a cut-off level of Bonferroni < 0.05 was used to determine statistical significance. A pilot study with a subset of these workers (*n* = 20) using the more limited Illumina HumanMethylation450 BeadChip array (Illumina, Inc., San Diego, CA, United States) for approximately 485,000 CpGs was published previously (Nylander-French et al., 2014). This study expands the number of workers by twenty percent and nearly doubles the number of CpGs that were evaluated (1.8 times more CpGs).

### Linear Mixed-Effects Models

Significant CpGs (*p* < 0.05) identified by the LR tests for the EWAS were further evaluated using mixed models. The mixed models in Statistical Analysis System (SAS) 9.4 (SAS Institute, 2017) have the advantage of being able to use the repeated measurements for the workers whose exposure was monitored for two or more workdays (“proc mixed” command with repeated measures), instead of only one summary value across multiple visits (as was used in R for the LR tests). CpG beta values were binned into three groups (low, medium, high methylation) with equal intervals for the mixed models using the “cut_interval” command in R, and all exposure and biomarker levels were natural log-transformed to improve normality and stabilize the variance. The mixed models included inhalation exposure, skin exposure, age, current smoking status, and ethnicity as covariates.

### Combining EWAS and GWAS Markers

Twenty SNPs correlated with isocyanate biomarker variability (false discovery rate, FDR < 0.05) were published previously for 33 workers (Taylor et al., 2020). Epigenetic data was successfully obtained for 24 individuals from that worker cohort. In order to make the results from the studies more comparable, PLINK v2.0, an open source genome association analysis toolset (Chang et al., 2015), was used to re-run the GWAS with the same 24 workers as the EWAS, using the same GWAS methods that were described previously (Taylor et al., 2020). The restricted GWAS with 24 workers resulted in 39 significant SNPs (Bonferroni < 0.05). Next, we determined whether there were significant SNPs from the full or restricted GWAS that were within 1 megabase pairs of the significant CpGs that were identified in the EWAS, and then SNPs that were proximal to the significant CpGs were added to the LR test in R as another covariate. CpG methylation has been shown to have long-range correlations with chromatin structure in the range of tens of kilobases (Zhang et al., 2017). The effect range that CpG methylation can have on gene expression is still largely unknown though, so a range of 1 megabase was chosen to decrease the chance of missing a meaningful interaction between a significant CpG site and significant SNP in a way that is associated with isocyanate biomarker levels.

### Bioinformatics

The potential biological relevance of the significant CpGs was evaluated through bioinformatic assessment. Genes in which the promoter or gene body contains the significant CpGs (based on hg38 annotations^3^, Zhou et al., 2017) were researched using NCBI PubMed, and the protein networks for the genes were evaluated using GeneMANIA, which has a label propagation algorithm to predict direct and indirect network interactions based off of validated unbiased protein-protein and protein-DNA interactions (Warde-Farley et al., 2010; Zuberi et al., 2013; Franz et al., 2018). We used GeneMANIA to learn more about co-expression, co-localization, genetic interactions, pathways, physical interactions, predicted networks, and shared protein domains in humans using the molecular function setting for the gene ontology weighting. The default settings were used for maximum resultant genes (*n* = 20) and the maximum resultant attributes (*n* = 10). A combined gene list for genes associated with the significant CpGs from the EWAS and the significant SNPs from our previously published GWAS was also input into GeneMANIA for bioinformatic assessment of the overlapping protein networks. The Database for Annotation, Visualization and Integrated Discovery (DAVID) v6.8 was also used to better understand the functions of the genes (Sherman et al., 2007).

## Results

### Epigenome-Wide Association Study

**FIGURE 1:**
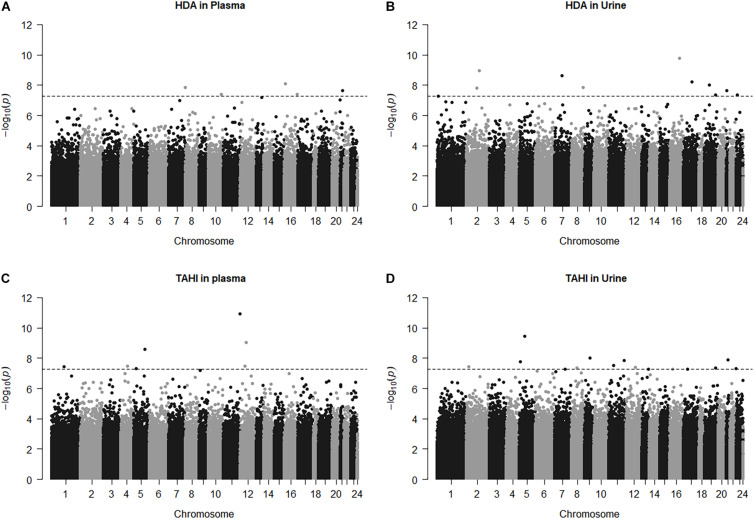
Manhattan plots for the model: biomarker level = inhalation exposure + skin exposure + age + smoking + ethnicity + CpG methylation level. The horizontal dashed line for significance is at *p* = 5.5E-8, which is approximately equivalent to Bonferroni < 0.05 for this study. CpG marker log_10_(*p*-values) are shown by chromosome for 1,6-diaminohexane (HDA) in plasma **(A)** and urine **(B)** and trisaminohexyl isocyanurate (TAHI) levels in plasma **(C)** and urine **(D)**.

Manhattan plots show that 38 CpGs were significantly associated with differences in biomarker levels in the exposed workers (Bonferroni < 0.05, Figure 1). Five CpGs were associated with plasma HDA levels, eleven were associated with urine HDA levels, seven were associated with plasma TAHI levels, and fifteen were associated with urine TAHI levels (Table 2). There were 13 CpGs that had low methylation levels, with betas < 0.50 (average beta of 0.05), and 25 CpGs that had high methylation levels with nearly all betas > 0.50 (average beta of 0.90) (data available upon request). The mean difference between the highest and lowest beta for all 38 CpGs was 0.095 (which is a 9.5% average variation in methylation level). Dose-response trends were observed for the significant CpGs; for 15 CpGs, workers with higher methylation levels had higher biomarker levels, and for the other 23 CpGs, workers with higher methylation levels had lower biomarker levels (Supplementary Figure 1). Annotations for the 38 significant CpG markers included 18 genes: ALG1, ANKRD11, C16orf89, CHD7, COL27A, FUZ, FZD9, HMGN1, KRT6A, LEPR, MAPK10, MED25, NOSIP, PKD1, SNX19, UNC13A, UROS, and ZFHX3.

**TABLE 2:** Significant CpGs (Bonferroni < 0.05) for the EWAS likelihood ratio (LR) tests with inhalation exposure, skin exposure, age, smoking, and ethnicity included as covariates. Annotations are based on the gene annotation file for the EPIC array available from http://zwdzwd.github.io/InfiniumAnnotation (Zhou et al., 2017), which was based on GENCODE v37 that was carried out on genome assembly GRCh38 (hg38). Significant CpGs are shown for 1,6-diaminohexane (HDA) levels in plasma **(A)** and urine **(B)** and trisaminohexyl isocyanurate (TAHI) levels in plasma **(C)** and urine **(D)**.

CpG	CHR	Position	Location	Gene	Gene Transcript type(s)	Location of CpG in gene(s)	Unadj	FDR	Bonf
**(A) HDA plasma levels**									
Cg04623960	16	2112917	S_Shore	PKD1	Protein coding	Gene body	8.0E-09	5.9E-03	6.9E-03
cg27127351	8	720292	–	ERICH1	Protein coding	Gene body	1.4E-08	5.9E-03	1.2E-02
cg14192762	21	39349758	S_Shore	HMGN1	Protein coding	Gene promoter	2.4E-08	6.8E-03	2.0E-02
cg25974451	10	125816801	–	UROS	Protein coding	Gene body	3.9E-08	7.1E-03	3.4E-02
cg16623734	16	89383614	–	ANKRD11	Protein coding	Gene body	4.1E-08	7.1E-03	3.5E-02
**(B) HDA urine levels**									
cg00979438	16	73165945	–	ZFHX3	Protein coding [transcription factor]	Gene body	1.7E-10	1.5E-04	1.5E-04
cg25391315	2	137776115	–	–	–	–	1.1E-09	4.6E-04	9.3E-04
cg01445942	7	73433645	Island	FZD9	Protein coding	Gene promoter	2.4E-09	6.9E-04	2.1E-03
cg05828606	17	47403690	–	EFCAB13	Protein coding	Gene body	6.0E-09	1.3E-03	5.2E-03
cg02690797	19	17648826	S_Shore	UNC13A	Protein coding	Gene body	9.3E-09	1.6E-03	8.0E-03
cg05517610	8	141616492	N_Shelf	AC025839.1	lncRNA	Gene body	1.4E-08	1.9E-03	1.2E-02
cg27351274	2	107542785	N_Shore	LINC01885; LINC01886	lncRNA; lncRNA	Gene body; Gene body	1.6E-08	2.0E-03	1.4E-02
cg26289880	21	32279200	Island	MIS18A; MIS18A-AS1	Protein coding; lncRNA	Gene promoter; Gene promoter	2.2E-08	2.4E-03	1.9E-02
cg14895646	19	49818209	N_Shore	FUZ;MED25	Protein coding; Protein coding	Gene promoter; Gene promoter	4.4E-08	3.8E-03	3.8E-02
cg22847973	X	33725448	N_Shore	AL591378.1; AL591501.1	lncRNA; lncRNA	Gene promoter; Gene promoter	4.5E-08	3.8E-03	3.8E-02
cg00829990	1	3012399	N_Shore	–	–	–	5.1E-08	4.0E-03	4.4E-02
**(C) TAHI plasma levels**									
cg05962950	11	130916669	Island	SNX19	Protein coding	Gene promoter	1.2E-11	1.0E-05	1.0E-05
cg04318458	12	52492715	–	KRT6A	Protein coding	Gene promoter	8.9E-10	3.8E-04	7.7E-04
cg02395590	5	141821939	–	–	–	–	2.6E-09	7.6E-04	2.3E-03
cg10292482	12	47360868	–	LINC02416	lncRNA	Gene body	3.2E-08	5.4E-03	2.7E-02
cg22548790	4	86590285	N_Shelf	MAPK10	Protein coding	Gene body	3.4E-08	5.4E-03	2.9E-02
cg16265717	1	65527465	S_Shore	LEPR	Protein coding	Gene body	3.8E-08	5.4E-03	3.3E-02
cg13305473	5	34190708	–	AC138409.1; AC138409.2	Pseudogene; Pseudogene	Gene body; Gene body	5.1E-08	6.2E-03	4.4E-02
**(D) TAHI urine levels**									
cg08564172	5	74537986	–	LINC01331	lncRNA	Gene promoter	3.5E-10	3.0E-04	3.0E-04
cg13748564	9	114155112	Island	COL27A1	Protein coding	Gene promoter	1.0E-08	3.0E-03	8.7E-03
cg23531127	21	38917339	–	AP001042.1	lncRNA	Gene body	1.3E-08	3.0E-03	1.2E-02
cg05005343	11	122099576	–	MIR100HG	lncRNA	Gene body	1.4E-08	3.0E-03	1.2E-02
cg00842318	5	2642243	N_Shore	–	–	–	1.8E-08	3.0E-03	1.5E-02
cg20625767	11	33172607	–	CSTF3-AS1; Y_RNA	lncRNA; misc_RNA	Gene body; Gene promoter	3.1E-08	3.1E-03	2.7E-02
cg08072606	2	20657621	–	–	–	–	3.6E-08	3.1E-03	3.1E-02
cg24626795	12	94769152	–	–	–	–	3.9E-08	3.1E-03	3.4E-02
cg19926587	8	60740402	–	CHD7	Protein coding	Gene body	4.3E-08	3.1E-03	3.7E-02
cg21292909	19	49556679	Island	NOSIP	Protein coding	Gene body	4.5E-08	3.1E-03	3.9E-02
cg24139837	X	3812109	N_Shelf	–	–	–	4.7E-08	3.1E-03	4.0E-02
cg22957752	17	22454553	Island	–	–	–	5.1E-08	3.1E-03	4.4E-02
cg04533202	16	5055937	–	ALG1; C16orf89	Protein coding; Protein coding	Gene promoter; Gene body	5.2E-08	3.1E-03	4.5E-02
cg00950418	7	105388677	Island	SRPK2	Protein coding	Gene body	5.3E-08	3.1E-03	4.6E-02
cg10125399	13	112674664	–	ATP11AUN	lncRNA	Gene body	5.4E-08	3.1E-03	4.6E-02

*Bonf, Bonferroni adjusted *p*-value; CHR, chromosome; FDR, false discovery rate; N_shelf, north shelf; S_shelf, south shelf; Unadj, unadjusted *p*-value.*

### Linear Mixed-Effects Models

HDI monomer skin exposure was associated with urine HDA biomarker levels (*p* = 0.008) (see Supplementary Table 1). When the most significant CpG for each biomarker was added to the mixed models using repeated measures for the isocyanate exposure and biomarker levels, three of the four CpGs remained significant at a level of *p* < 0.05 (HDA plasma cg04623960, TAHI plasma cg05962950, TAHI urine cg08564172; see Supplementary Table 2), only the most significant CpG for HDA in urine (cg00979438) did not remain significant in the mixed models. Smoking and ethnicity were not significant in any of the mixed models (*p*-values for both > 0.2). Age (*p* = 0.04) and HDI monomer inhalation exposure (*p* = 0.02) were significant for HDA levels in plasma, and HDI monomer skin exposure (*p* = 0.02) was significant for HDA levels in urine. Skin exposure, inhalation exposure, age, smoking, nor ethnicity were significant in the mixed models for TAHI that included the most significant CpGs (Supplementary Table 2).

### Combining EWAS and GWAS Markers

When the locations of the SNPs for the published GWAS results (*n* = 33) were compared to the significant CpGs, none were observed to be <1 megabase apart. When the GWAS was re-run with only the 24 workers included in the EWAS, there was one SNP, rs3211803 (within the *F10* blood coagulation gene), that was 475,213 base pairs away from one of the CpGs associated with TAHI urine levels, cg10125399 (within the *ATP11AUN* gene associated with A1C levels, Soranzo et al., 2010). Adding genotype data for rs3211803 to the LR test evaluating the association between cg10125399 methylation and TAHI urine levels changed the *p*-value of the LR test from *p* = 5.4E-8 without the SNP to *p* = 2.0E-4 with the SNP included in the model. When the CpG was removed from the model to evaluate the *p*-value for the SNP association with TAHI urine biomarker levels separately, the *p*-value for the SNP was 3.3E-12.

### Bioinformatics

The genes that are associated with the 38 significant CpGs are listed in Table 2, and the functions of the proteins that are encoded by those genes are listed in Supplementary Table 3. Genes associated with the CpGs for plasma HDA levels included: *PKD1, HMGN1, UROS*, and *ANKRD11* (in order of significance). The listed NCBI functions for the PKD1 protein include regulation of calcium cation channels and intracellular calcium homeostasis, as well as involvement in cell–cell/matrix interactions, signal transduction modulation, and renal tubular development. HMGN1 binds to DNA for transcription regulation, UROS is involved in heme biosynthesis, and ANKRD11 inhibits ligand-dependent activation of transcription. Genes associated with the CpGs for urine HDA levels included *ZFHX3*, *FZD9, UNC13A* (also known as *Munc13-1*), *FUZ*, and *MED25*. NCBI listed functions for the protein products of those genes were that: ZFHX3 is a transcription factor that regulates myogenic and neuronal differentiation, FZD9 is a receptor for Wnt signaling proteins, UNC13A binds to phorbol esters and diacylglycerol (DAG) to impact neurotransmitter release at synapses, FUZ is involved in ciliogenesis and directional cell movement, and MED25 is a component of a transcriptional coactivation complex that is required in for transcribing most RNA polymerase II-dependent genes and is involved in chromatin modification.

The genes associated with the CpGs that were significant for variability in plasma TAHI levels included: *SNX19, KRT6A, MAPK10*, and *LEPR*. The NCBI functions for the protein products of those genes were that: SNX19 is involved in insulin section, KRT6A is a member of the keratin gene family that is expressed in simple and stratified epithelial tissues, MAPK10 is a mitogen-activated protein kinase that may play a role in stress-induced neuronal apoptosis, and LEPR is a receptor for leptin and is important for the regulation of fat metabolism as well as regulation of lymphopoiesis (the generation of lymphocytes). The genes associated with the CpGs that were significant for variability in urine TAHI levels included *COL27A, CHD7, NOSIP, ALG1*, and *C16orf89*. The NCBI listed functions include that: COL27A impacts tissue growth and repair for calcification of cartilage and impacts the transition of cartilage to bone, CHD7 is a chromodomain helicase DNA binding protein, nitric oxide synthase interacting protein (NOSIP) is involved in modulation of nitric oxide synthase protein activity, ALG1 catalyzes the first mannosylation step in the biosynthesis of lipid-linked oligosaccharides, and C16orf89 is expressed in the thyroid and may play a role in thyroid function and development.

**FIGURE 2:**
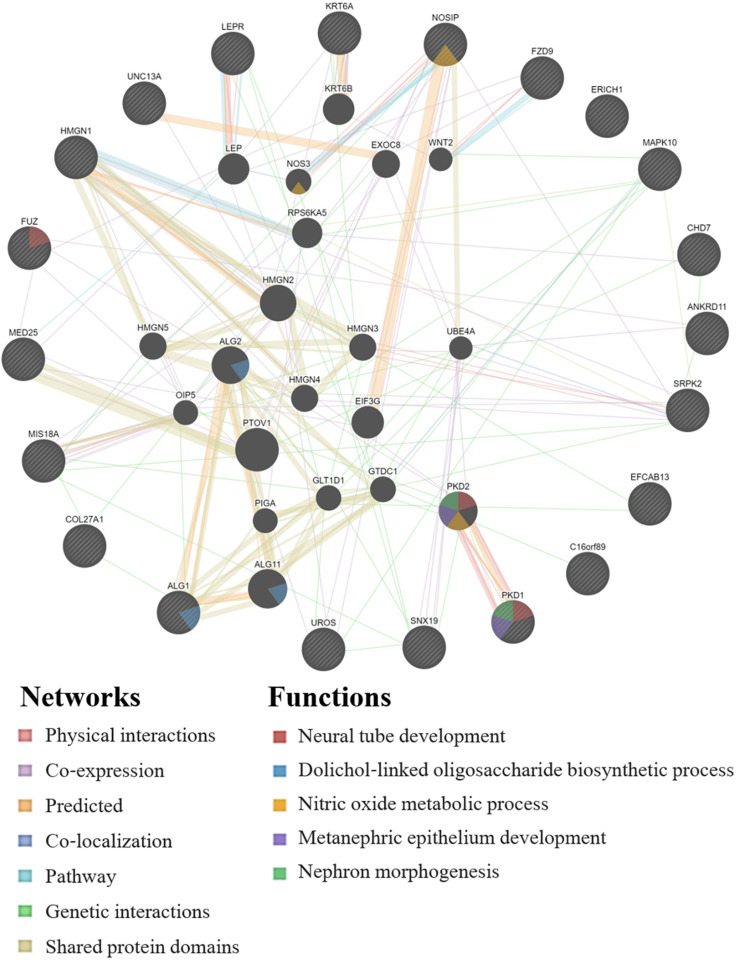
GeneMANIA network for the genes associated with the EWAS significant CpGs for the model: biomarker level = inhalation exposure + skin exposure + age + smoking + ethnicity + CpG methylation level. Query genes have black circles with white-striped lines, the networks are shown with the colored lines between genes, and the shading on the circles shows the functions.

**FIGURE 3:**
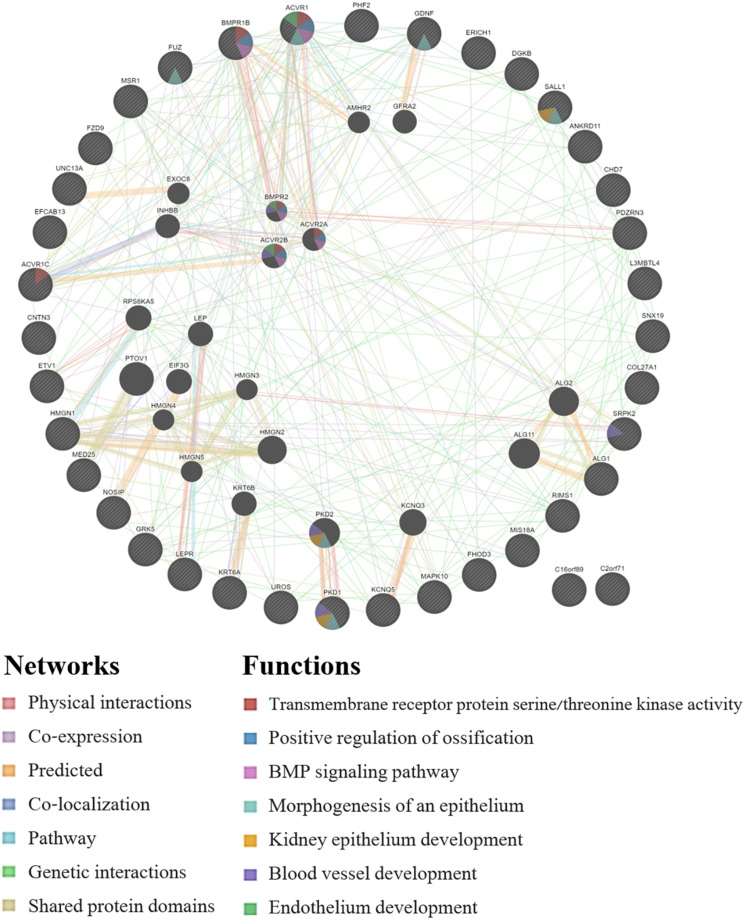
GeneMANIA network for the genes that were associated with the EWAS (*n* = 24 workers) and the full GWAS (*n* = 33 workers). Query genes have black circles with white-striped lines, the networks are shown with the colored lines between genes, and the shading on the circles shows the functions.

GeneMANIA analysis for the EWAS associated genes above shows protein network functions that include neural tube development, dolichol-linked oligosaccharide biosynthetic process, nitric oxide metabolic process, metanephric epithelium development, and nephron morphogenesis (see Figure 2; full GeneMANIA reports available upon request). When the genes from the EWAS and the previously published GWAS (Taylor et al., 2020) were input into GeneMANIA, the protein functions identified included transmembrane receptor protein serine/threonine kinase activity, positive regulation of ossification, bone morphogenic protein (BMP) signaling pathway, morphogenesis of an epithelium, kidney epithelium development, blood vessel development, and endothelium development (see Figure 3). There were genetic interactions between almost all of the input genes, except for two open reading frame genes (C16orf89 and C2orf71).

## Discussion

### Epigenome-Wide Association Study

We observed 38 CpGs with dose-response trends for differences in plasma and urine biomarker levels in autobody spray-painters occupationally exposed to isocyanates. For 15 CpGs, workers with higher methylation levels had higher biomarker levels, and for the other 23 CpGs, workers with higher methylation levels had lower biomarker levels. If the methylation patterns found for these CpGs are confirmed by other studies, then further research is needed to determine whether transcription is being affected. If increased or decreased transcription of any of the genes associated with the significant CpGs is identified, then those protein networks can be investigated more in order to verify whether they contribute to altering the toxicokinetics of isocyanates.

### Linear Mixed-Effects Models

When the most significant CpG for each biomarker was added to mixed models with the repeated exposure and biomarker level measurements, most of the CpGs remained significant (*p* < 0.05). Smoking and ethnicity were not significantly associated with any of the biomarker levels in the mixed models (*p*-values for both > 0.2), which suggests that smoking and ethnicity do not have a meaningful impact on the toxicokinetics of isocyanates, or it could be an artifact of our small sample size, so it needs to be studied further with a larger study population. Age was only significant in the model for HDA plasma levels (*p* = 0.04) and was insignificant in the other three models. This indicates that age, smoking, and ethnicity had little impact on the observed biomarker levels in this study. However, the variables were left in the models as a precaution because age, smoking, and ethnicity are known to be able to impact epigenetic DNA methylation levels.

The relationship between isocyanate inhalation and skin exposure and the isocyanate biomarker levels was also evaluated with the mixed models. As was the case for the GWAS with 33 workers (Taylor et al., 2020), the EWAS mixed models with 24 of these workers still showed that HDI monomer exposure through both inhalation and skin was significantly associated with the biomarker levels; HDI monomer inhalation exposure was associated with HDA plasma biomarker levels (*p* = 0.02), and HDI monomer skin exposure was associated with HDA urine biomarker levels (*p* = 0.02). Inhalation exposure nor skin exposure to HDI isocyanurate were significant in the models for TAHI in plasma or urine, which could be because the biomarker sample collections were optimized for the 3 h half-life of HDA in urine (Gaines et al., 2010a) and it might not be the ideal sampling schedule for detecting TAHI in plasma or urine. Because inhalation and skin exposure were both significant for HDA biomarker levels, this indicates that industrial hygiene measures should be taken to prevent exposure to isocyanates not just through respiratory protection, but through skin protection measures as well.

### Combining EWAS and GWAS Markers

One significant SNP from the restricted GWAS (*n* = 24), rs3211803, was observed to be about half a megabase away from one of the CpGs, cg10125399, that was associated with TAHI biomarker levels. We tested whether there is a mediation effect of the SNP on the CpG by adding the SNP to the likelihood ratio test model for the CpG’s association with TAHI urine levels (with inhalation exposure, skin exposure, age, ethnicity, and smoking included as covariates). Instead of strengthening the model, we observed that the *p*-value became four orders of magnitude weaker when the SNP was added to the model (CpG *p* = 5.6E-08 without the SNP vs. *p* = 2.0E-04 with the SNP). This implies that the SNP and CpG marker are not independent of each other, potentially due to the SNP rs3211803 mediating the methylation level at the CpG site cg10125399, and therefore, rs3211803 and cg10125399 should not be used in the same model to explain the TAHI urine biomarker level. When evaluating which marker better predicts TAHI urine biomarker level, the *p*-value was observed to be strongest when the rs3211803 SNP genotype was used for the likelihood ratio test instead of the cg10125399 CpG (SNP *p* = 3.3E-12), indicating that genotyping rs3211803 is more predictive for TAHI biomarker levels than determining the methylation level of cg10125399.

### Bioinformatics

The specific genes associated with the significant CpGs were all novel and did not overlap with the genes that have previously been reported in human studies to be associated with isocyanate biomarker levels or isocyanate-induced health effects. A list of previously reported isocyanate-asthma related genes for this evaluation can be found in our GWAS paper (Taylor et al., 2020). Even though none of the genes were the same, there was overlap in the gene functions for the EWAS biomarker research with the GWAS biomarker research and isocyanate-asthma research, including: cell migration, cell–cell adhesions, calcium cation channels and calcium regulation, transcription regulation, angiogenesis, apoptosis regulation, and nitric oxide production.

One of the potentially meaningful protein pathways was keratinocyte cell migration and sebaceous gland morphogenesis, in which a CpG that was significantly associated with levels of HDA in urine is within the gene keratin 6A (*KRT6A*). KRT6A regulates keratinocyte migration by altering cell–cell adhesions and cell–matrix adhesions (Wang et al., 2018). KRT6A has also been shown to be involved in sebaceous gland morphogenesis in skin (Gu and Coulombe, 2008). Differences in duct fate could be of particular importance for isocyanates considering that isocyanates have been shown to enter the skin through hair follicles and sebaceous glands (Nayak et al., 2014). This suggests that changes in *KRT6A* expression have the potential to impact the skin barrier layer. Variation in the effectiveness of the skin barrier could result in inter-individual differences in the absorption of isocyanates through skin and into the body, and thus result in differences in biomarker levels amongst workers with similar exposure levels.

Additionally, mitogen-activated protein kinase (*MAPK*) and apoptosis regulation overlap with previous isocyanate research. A CpG that was significant in this EWAS for differences in TAHI plasma levels is within *MAPK10*, a stress-induced neuronal apoptosis gene (RefSeq, 2017). The relationship between TDI and *MAPK* genes has been studied previously *in vitro* with mixed results. Kim et al. (2010) observed that TDI suppressed the activity of MAPKs in a dose-dependent manner, including p38 MAPK, but a recent study by Wang et al. (2017) showed that TDI-human serum albumin (TDI-HSA) conjugates activated p38 MAPK signals. Wang et al. (2017) further demonstrated that the activation of MAPK by TDI-HSA was mitigated by pre-treating cells with short thymic stromal lymphopoietin (short TSLP), which is thought to have an anti-inflammatory function. The discrepancy in the findings by Kim et al. (2010) and Wang et al. (2017) might be partly because the studies used different human bronchial epithelial cell lines [Kim et al. (2010) used A549 cells and Wang et al. (2017) used 16HBE cells] and/or because Kim and collegues exposed cells to TDI whereas Wang and collegues exposed cells to TDI-HSA, which may have changed the reactivity of the TDI NCO groups (Hettick et al., 2009). In isocyanate asthma research, another apoptosis regulating gene, *LHPP*, had a significant SNP that was associated with isocyanate-asthma risk (Kim et al., 2009) and a SNP within *LHPP* was significant in the expanded candidate-gene analysis for association with isocyanate biomarker levels in our GWAS (Taylor et al., 2020). There was also a significant SNP within *GDNF* for HDA plasma levels (Taylor et al., 2020), and GDNF has also been linked to regulation of apoptosis (Zhang and Wang, 2019). Together, this provides additional evidence that the role of MAPKs and apoptosis in cellular response to isocyanate exposure should be studied more.

One way that differences in apoptosis regulation could potentially impact isocyanate biomarker levels is indirectly through causing differences in levels of apoptosis compared to other forms of cell death, and consequently, differences in inflammatory responses. Cell death mechanisms are highly regulated and have important implications for the resulting inflammation levels (Davidovich et al., 2014; Messer, 2017; Vasilikos et al., 2017). For example, apoptosis is thought to result in little if any inflammation, whereas necrosis and necroptosis are pro-inflammatory (Davidovich et al., 2014). This may then impact the distribution of chemicals in the body; multiple studies have shown that inflammation increases the uptake of drugs into inflamed tissue (Vugmeyster et al., 2012). This appears to be because inflammation results in increased vascular permeability and weakened endothelial barriers, which is meant to enable white blood cells to more easily migrate from the blood and into inflamed tissues, but that may enable chemicals to cross through blood vessels into tissues more easily as well (Vugmeyster et al., 2012; Mittal et al., 2014), particularly in the case of chronic exposure. Therefore, differences in regulation of apoptosis could modulate how much or how little inflammation results after exposure to isocyanates and thus impact isocyanate biomarker levels indirectly by changing the uptake of isocyanates into inflamed tissues.

Angiogenesis might also play a role in isocyanate ADME. LEPN observed in this EWAS is involved in angiogenesis, PTGS1 from isocyanate-asthma research (Yucesoy et al., 2016) is involved in angiogenesis regulation (RefSeq, 2014), and SALL1 and PDZRN3 observed in our GWAS on isocyanate biomarkers (Taylor et al., 2020) are also involved in angiogenesis and vascular morphogenesis, respectively (Yamamoto et al., 2010; Sewduth et al., 2014). If there are differences in the expression of these genes that results in more or less formation of new blood vessels in some workers than others, then one way this might change biomarker levels is by impacting how isocyanates are moved through the body and potentially in what concentration levels. Angiogenesis is also a sign of inflammation (Mor et al., 2004) and may be associated with differences in isocyanate biomarker levels indirectly through association with differences in inflammatory responses in workers, which may then be impacting the absorption and distribution of isocyanates.

*ZFHX3 and FZD9* are two other genes associated with significant CpGs that have potentially important functions, including *ZFHX3*’s involvement in regulating both intracellular calcium homeostasis (Zhao et al., 2019) and mitochondrial oxidative stress (Lkhagva et al., 2021), and *FZD9*’s involvement in bone mineralization (Albers et al., 2011; Heilmann et al., 2013) and B-cell development (Ranheim et al., 2005). Bone mineralization is a pathway that also arose in our GWAS biomarker research (ACVR1, BMPRB) (Taylor et al., 2020) and has implications for calcium regulation. These two genes’ calcium regulation functions are notable because Chiung et al. (2010) demonstrated that calcium levels impact IL-4 cytokine production after isocyanate exposure. Therefore, differences in calcium regulation could potentially impact isocyanate biomarker levels by altering inflammation levels. The other function of FZD9 involving B-cell development could be more important for isocyanate toxicodynamics. In a mouse model study that included bone marrow reconstitution in a wild-type host, it was observed that FZD9 knock-out (FZD9–/–) resulted in abnormal B-cell development from defective signaling during the pre-B stage when the cells normally undergo self-renewal before further differentiation (Ranheim et al., 2005). Abnormalities in B-cell development could be meaningful for toxicodynamics of isocyanates in chronically exposed workers, considering that B cells are responsible for producing immunoglobulin E (IgE) antibodies for isocyanates and a small subset of workers develop IgE against isocyanates (Jones et al., 2006; Fisseler-Eckhoff et al., 2011).

Nitric oxide signaling and calcium ion regulation pathways were also identified in this analysis. One CpG gene associated with variability in TAHI urine levels was *NOSIP*. Before asthma development, nitric oxide may be associated with isocyanate biomarker level variability indirectly due to its role in inflammation (Sharma et al., 2007). NOSIP affects the activity of endothelial nitric oxide synthase (eNOS) and neuronal nitric oxide synthase (nNOS) (Dedio et al., 2001; Dreyer et al., 2004). eNOS is produced by vascular epithelium and impacts vasorelaxation, cell proliferation, white blood cell adhesions, and platelet aggregation (Bignon et al., 2019). nNOS is important for neurotransmission and neuromodulation, and it is expressed in many parts of the body, including neurons, lung epithelial cells, kidneys, and skin. The activity of eNOS and nNOS is calcium-regulated (Kopincová et al., 2011), which is interesting because calcium cation channels and calcium regulation were pathways that arose in our GWAS isocyanate-biomarker research (Taylor et al., 2020). Additionally, one of the products of a gene that was proximal to a SNP that was associated with HDA urine biomarker levels, DGKB (Taylor et al., 2020), regulates diacylglycerol (DAG) levels, which increases protein kinase C (PKC) that impacts NOS production (Leppänen et al., 2014). UNC13A observed in the our EWAS also binds with DAG (RefSeq, 2012) and may alter NOS levels, and nitric oxide production by NOS plays a role in apoptosis and bone resorption (Sharma et al., 2007). Recently, a study with *in vivo* and *in vitro* components was able to elucidate that exposure to an isocyanate that is related to HDI, MDI, activated inducible NOS (iNOS) transcription through calcineurin/NFAT signaling (Lin et al., 2020). Calcineurin is a calcium and calmodulin dependent serine/threonine protein phosphatase that is important for calcium-mediated signal transduction pathways (Liu et al., 2005). Furthermore, the importance of nitric oxide has been demonstrated in many human studies on asthma (Allmers et al., 2000; Barbinova and Baur, 2006; Ferrazzoni et al., 2009; Pedrosa et al., 2012; Jonaid et al., 2014; Mason et al., 2016; Engel et al., 2018; Yatera and Mukae, 2019). These studies have revealed that a substantial increase in exhaled nitric oxide the day after specific-inhalation challenge to isocyanates or other allergens was indicative of a patient having asthma. An *in vivo* study has even demonstrated that mice without NOS isoforms (n/i/eNOS^–/–^) did not develop markers of asthma (Yatera and Mukae, 2019).

The association of NOSIP and DGKB with biomarker levels of the workers in our study provides novel evidence that NOS and nitric oxide may be impacting the toxicokinetics of isocyanates even before asthma development. The overlap in human biomarker research pathways and in human, *in vivo*, and *in vitro* isocyanate-asthma research provides compelling evidence that NOS activity and nitric oxide signaling are important to isocyanate ADME and, thus, may contribute to the pathogenesis of isocyanate-induced asthma.

### Limitations

The greatest limitation of this study is the small exposed population sample size. There is also the potential presence of false positive and/or false negative results, so all findings should be followed-up with additional studies on a larger population sample to investigate both genetic and epigenetic signatures affecting biomarkers of exposure and isocyanate induced asthma. The use of continuous quantitative data (instead of categorical data) for the exposure levels, biomarker levels, and methylation levels enabled us to identify statistically significant CpG sites, but those measurements may differ from the true values. For the exposure measurements, an over- or under-estimation of the workers’ exposures may have occurred due to the use of personal protective equipment (PPE). HDI isocyanurate skin exposure is more subject to under-estimation than HDI monomer skin exposure because HDI isocyanurate is absorbed more quickly into the skin (Thomasen and Nylander-French, 2012) and, thus, the tape-strip sampling results may have had greater impact on under-estimating HDI isocyanurate dose compared to HDI monomer dose. Additionally, worker’s inhalation exposure levels were adjusted for both the type of respiratory protection worn and the average breathing rate for an average adult male worker. Therefore, the true level of an individual worker’s exposure may vary from our estimates despite the personal quantitative exposure measurements.

Biomarker levels may also differ from the exposure the workers received that day. We were able to collect worker samples only during the workday; being able to extend the biomonitoring timeframe would be ideal. Another limitation is that there may have been carry-over of HDA and/or TAHI from previous work-day exposures. For TAHI biomarker levels, a notable limitation is that only a few workers had detectable levels of TAHI. Detection of TAHI being less frequent than HDA may be due to multiple factors, such as the timing of the sample collection (which was optimized for the 3-h half-life of HDA), the potential for differences in their main routes of excretion (which is unknown for HDI and its oligomers), and because the workers were using PPE that may have altered exposure to HDI isocyanurate oligomer differently than for HDI monomer. For example, 3M respirators provide a higher workplace protection factor for HDI oligomers than for HDI monomer (Liu et al., 2006). Lastly, the epigenetic DNA methylation levels were only evaluated for PBMCs as a sentinel cell type, in which PBMCs were chosen due to their long survival time in circulation and their exposure to isocyanates that enter the body from both inhalation and skin exposure. However, it is notable that methylation levels vary throughout the body by cell type. Additionally, we used only one blood sample collected from each worker to determine the workers’ epigenetic signatures. Little is known about the variability of methylation levels over time in this worker population, so workers’ methylation levels could have differed in the samples collected during the other work visits that were not evaluated for methylation levels.

For future research, increasing the sample size of exposed workers, extending the timeframe for biomarker sampling by an additional 24–72 h, and measuring methylation at multiple time points could increase the study power, help to detect HDA and TAHI in workers’ plasma and urine, and aid in identification and confirmation of CpG sites that are associated with isocyanate exposure biomarker levels.

### Strengths

Two main strengths of this study were the extensive exposure and biomonitoring data that was collected and the strict QC measures that were implemented for the epigenetics data. We used repeated personal quantitative measurements to identify and combine epigenetic and genetic markers associated with isocyanate exposure and biomarkers of exposure. The workers were monitored repeatedly over time (2–3 times each) to better account for inter- and intra-individual variability and, thus, increase the reliability of the industrial hygiene data (Rappaport et al., 1995; Lyles et al., 1997; Serdar et al., 2006). Another advantage was that the biomarkers of exposure that were measured in this study are derived from the parent isocyanates and are thus specific for the exposure (i.e., no other chemical exposure or metabolic process could confound the biomarker measures). Furthermore, the only isocyanates that the workers were occupationally exposed to were HDI and its oligomers, meaning that there is no confounding from exposure to related but structurally different isocyanates such as TDI or MDI, which might have different ADME than HDI monomer and HDI isocyanurate. For the methylation levels, strict QC measures were used during preprocessing in order to reduce the inclusion of unreliable measurements for CpGs, including using Enmix and RCP normalization methods and a strict cut-off (*p* = 1E-6) for the detection *p*-values in order for a beta data point to be included, all of which reduces the inclusion of data with low intra-class correlation coefficient values (Xu and Taylor, 2020). Regarding worker health status, workers with asthma or smoking in their history were included because the aim of this study is to examine how differences in methylation levels are correlated with differences observed in worker biomarker levels, regardless of what caused the differences in the workers’ methylation levels. Thus, differences in workers’ health status might impact worker DNA methylation levels in a manner that actively helps to better inform our biomarker models.

Another strength of this study is the bioinformatics assessment methods and the prospective exploratory nature of EWAS, which is not limited by previous hypotheses. Bioinformatics analysis of the functions of the proteins for the genes with significant CpGs was aided by the use of GeneMANIA and resulted in finding protein pathways that overlap with previous isocyanate research. If only the list of genes with significant CpGs was compared to the list of genes with previously reported epi/genetic marker associations with isocyanate biomarker levels and/or isocyanate-asthma, then no overlap would have been identified even though the protein pathway functions have a substantial amount of overlap. This demonstrates the importance of evaluating the protein networks associated with significantly associated genes when assessing the biological relevance and overlap in epi/genetic studies.

One of the most unique aspects of this study is that we modeled continuous data for all workers without having to separate them into binary groups, such as controls vs. non-controls or low vs. high biomarker levels. Exposed workers in this study population had anywhere between undetectable levels of isocyanate biomarkers in their plasma and urine, to levels that were much higher than the average of their peers. That made it very powerful to be able to model the full continuous data set without having to arbitrarily bin workers into different groups such as for exposure level, biomarker level, or DNA methylation level. Additionally, unexposed individuals would not add any value to our study because people who are not exposed to isocyanates will have no isocyanate biomarkers in their plasma or urine, meaning that their methylation data cannot be modeled for associations with post-exposure biomarker level variability. Future studies can explore if the isocyanates may have caused the differences in worker DNA methylation levels by studying whether the workers’ methylation profiles differ from unexposed individuals, but that was outside of the scope of this project. The strength of this study is to illustrate the impact of variation in methylation levels on isocyanate toxicokinetics, regardless of what originally caused that methylation variation among these chronically exposed workers.

## Conclusion

Epigenetic markers, as with genetic markers, are associated with inter-individual differences in workers’ biomarker levels after exposure to HDI monomer and HDI isocyanurate. The observed genes associated with the significant CpGs revealed protein network functions including: cell–cell adhesions, keratinocyte cell migration, as well as regulation of transcription, apoptosis, nitric oxide production, and calcium transport. These functions could potentially impact biomarker levels by changing how readily isocyanates are absorbed into the body (via differences in cell–cell adhesions in skin), how the isocyanates are then distributed in the body due to differences in how much inflammation the isocyanates cause (via differences in the regulation of apoptosis and nitric oxide production), and how the isocyanates are transported within the body for excretion (via differences in the regulation of transport channels). Further research is needed to determine whether the associated methylation differences at the CpG sites are impacting biomarker levels through causing changes in transcription levels. This knowledge can help guide research on the mechanism of isocyanate-induced medical illnesses including asthma and skin reactivity.

## Data Availability Statement

The datasets for this article are not publicly available due to concerns regarding participant/patient anonymity. Requests to access the datasets should be directed to the corresponding author.

## Ethics Statement

The studies involving human participants were reviewed and approved by the University of North Carolina at Chapel Hill Office of Human Research Ethics Institutional Review Board (Study #12-1195) and Washington University Department of Social and Health Services Institutional Review Board (Study #A-013106). The participants provided their written informed consent to participate in this study.

## Author Contributions

LT designed and carried out the model computations, created the figures, performed the bioinformatics analyses, and prepared the manuscript. JF provided the guidance for the genetics and bioinformatics methods. ZR conducted the quantitative analysis of the exposure and biomarker levels. LN-F designed and supervised the project. All authors contributed to and approved the final manuscript.

## Conflict of Interest

The authors declare that the research was conducted in the absence of any commercial or financial relationships that could be construed as a potential conflict of interest.

## Footnotes

1 http://cran.r-project.org/src/base/R-4/R-4.0.1.tar.gz

2 https://cran.r-project.org/web/packages/lmtest/lmtest.pdf

3 http://zwdzwd.github.io/InfiniumAnnotation

## Abbreviations

ADME: absorption, distribution, metabolism, and elimination
APF: assigned protection factor
BMI: body-mass index
BSA: body surface area
CpG: cytosine and guanine connected by a phosphodiester bond
DAVID: database for annotation, visualization, and integrated discovery
EWAS: epigenome-wide association study
FDR: false discovery rate
GC-MS: gaschromatography mass-spectrometry
GWAS: genome-wide association study
HDA: 1,6-hexamethylene diamine
HDI: 1,6-hexamethylene diisocyanate
HPLC-MS: high-performance liquid chromatography-mass spectrometry
LD: Linkage disequilibrium
LR: likelihood ratio
LOD: limit of detection
MDI: methylene diphenyl diisocyanate
MDL: method detection limit
nano-UPLC-ESI-MS/MS: nanoflow ultra-performance liquid chromatography nano-electrospray ionization tandem mass spectrometry
NCBI: National Center for Biotechnology Information
OSHA: Occupational Safety and Health Administration
PBMC: peripheral blood mononuclear blood cells
PPE: personal protective equipment
QC: quality control
RELIC: REgression on Logarithm of Internal Control
rsID: reference SNP cluster identification
SNP: single nucleotide polymorphism
STD: standard deviation
TAHI: trisaminohexyl isocyanurate
TDI: toluene diisocyanate.
