# Supplementary material for: Epigenetic Markers Are Associated With Differences in Isocyanate Biomarker Levels in Exposed Spray-Painters
Source: Front Genet. 2021 Jul 14;12:700636. doi: 10.3389/fgene.2021.700636 (PMC8318037; doi:10.3389/fgene.2021.700636)
Supplement: Supplementary file 1 [file Data_Sheet_1.pdf]

Supplemental Tables and Figures

Summary:

The Supplemental Tables S1 and S2 show the mixed models for HDA and TAHI levels with and without the most significant CpG added to the model, respectively. Supplemental Table S3 provides the NCBI descriptions for the genes associated with the significant CpGs. Supplemental Figure S1 shows the CpG methylation *vs.* biomarker level level plots.

Abbreviations:

CHR, chromosome;

CpG, cytosine and guanine connected by a phosphodiester bond;

EWAS, epigenome-wide association study;

GWAS, genome-wide association study;

HDA, 1,6-hexamethylene diamine;

HDI, 1,6-hexamethylene diisocyanate;

ISO, hexamethylene diiscoyanate isocyanurate;

NCBI, National Center for Biotechnology Information;

rsID, reference SNP cluster identification;

SNP, single nucleotide polymorphism;

TAHI, trisaminohexyl isocyanurate.

Supplemental Table S1. Mixed model results when using repeated measures for exposure and biomarker data from multiple visits. For smoking, non-smokers were coded as 0 and current smokers were coded as 1. For ethnicity, non-Latino whites were coded as 0 and other ethnicities were coded as 1.

1A) HDA levels in plasma

Effect	Estimate	Standard Error	p-Value
Intercept	-4.4440	1.5147	0.0071
HDI Inhalation Exposure	0.1550	0.1596	0.3366
HDI Skin Exposure	0.1156	0.0641	0.0784
Age	0.0392	0.0340	0.2606
Smoking	0.6207	0.3895	0.1236
Ethnicity	0.5281	0.8129	0.5218

1B) HDA levels in urine

Effect	Estimate	Standard Error	p-Value
Intercept	-4.4044	1.6145	0.0113
ISO Inhalation Exposure	-0.0175	0.1488	0.9067
ISO Skin Exposure	0.1513	0.0547	0.0081
Age	0.0307	0.0359	0.3968
Smoking Status	0.0662	0.3993	0.8696
Ethnicity	0.3414	0.8378	0.6869

1C) TAHI levels in plasma

Effect	Estimate	Standard Error	p-Value
Intercept	-6.1997	1.3753	0.0001
ISO Inhalation Exposure	0.0881	0.1129	0.4393
ISO Skin Exposure	0.1082	0.0845	0.2073
Age	-0.0374	0.0316	0.2477
Smoking Status	-0.1956	0.3688	0.6005
Ethnicity	0.5820	0.7609	0.4515

1D) TAHI levels in urine

Effect	Estimate	Standard Error	p-Value
Intercept	-8.0924	1.3876	<0.0001
ISO Inhalation Exposure	0.1995	0.1352	0.1468
ISO Skin Exposure	0.1318	0.08684	0.1357
Age	-0.01079	0.03302	0.7453
Smoking Status	0.0362	0.3711	0.923
Ethnicity	-0.8301	0.7656	0.2882

Supplemental Table S2. Mixed model results with the most significant CpG when using repeated measures for exposure and biomarker data from multiple visits. For smoking status, non-smokers were coded as 0 and current smokers were coded as 1. For ethnicity, non-Latino whites were coded as 0 and other ethnicities were coded as 1.

2A) HDA levels in plasma

Effect	Estimate	Standard Error	p-Value
Intercept	-0.4494	1.6363	0.7863
HDI Inhalation Exposure	0.3436	0.1386	0.0176
HDI Skin Exposure	0.0312	0.0663	0.6410
cg04623960 [0.904,0.919]	0	.	.
cg04623960 (0.919,0.934]	-1.8434	1.3937	0.2002
cg04623960 (0.934,0.949]	-4.6124	1.1878	0.0009
Age	0.0622	0.0289	0.0432
Smoking Status	-0.1439	0.3663	0.6985
Ethnicity	-0.1921	0.6606	0.7741

2B) HDA levels in urine

Effect	Estimate	Standard Error	p-Value
Intercept	-1.2851	2.2454	0.5742
HDI Inhalation Exposure	-0.0404	0.1678	0.8113
HDI Skin Exposure	0.1553	0.0630	0.0191
cg00979438 [0.665,0.769]	0	.	.
cg00979438 (0.873,0.978]	-2.2868	1.3892	0.1171
Age	0.0084	0.0434	0.8472
Smoking Status	-0.1607	0.4983	0.7508
Ethnicity	0.1950	1.0671	0.8571

2C) TAHI levels in plasma

Effect	Estimate	Standard Error	p-Value
Intercept	-7.9625	1.1352	<0.0001
ISO Inhalation Exposure	0.0457	0.0839	0.5899
ISO Skin Exposure	0.0091	0.0643	0.8878
cg05962950 [0.0099,0.0259]	0	.	.
cg05962950 (0.0259,0.0418]	-0.5053	0.7816	0.5261
cg05962950 (0.0418,0.0578]	4.7436	1.1390	0.0006
Age	0.0018	0.0231	0.9377
Smoking Status	0.3140	0.2431	0.2129
Ethnicity	0.2147	0.5300	0.6902

2D) TAHI levels in urine

Effect	Estimate	Standard Error	p-Value
Intercept	-9.0574	1.4196	<0.0001
ISO Inhalation Exposure	0.0876	0.1370	0.5258
ISO Skin Exposure	0.1682	0.0908	0.0707
cg08564172 [0.0263,0.0476]	0	.	.
cg08564172 (0.0476,0.0688]	-1.0216	0.7579	0.1908
cg08564172 (0.0688,0.0901]	2.5149	1.1722	0.0427
Age	0.0117	0.0344	0.7355
Smoking Status	0.2920	0.3799	0.4500
Ethnicity	-0.4195	0.7228	0.5673

Supplemental Table S3. Isocyanate biomarker-associated CpG genes that have descriptions on NCBI.

3A) HDA levels in plasma

CHR	Gene	Full Gene Name	Gene Function
16	PKD1	polycystin 1, transient receptor potential channel interacting	This gene encodes a member of the polycystin protein family. The encoded glycoprotein contains a large N-terminal extracellular region, multiple transmembrane domains and a cytoplasmic C-tail. It is an integral membrane protein that functions as a regulator of calcium permeable cation channels and intracellular calcium homoeostasis. It is also involved in cell-cell/matrix interactions and may modulate G-protein-coupled signal-transduction pathways. It plays a role in renal tubular development, and mutations in this gene cause autosomal dominant polycystic kidney disease type 1 (ADPKD1). ADPKD1 is characterized by the growth of fluid-filled cysts that replace normal renal tissue and result in end-stage renal failure. Splice variants encoding different isoforms have been noted for this gene. Also, six pseudogenes, closely linked in a known duplicated region on chromosome 16p, have been described. [provided by RefSeq, Oct 2008]
21	HMGN1	high mobility group nucleosome binding domain	The protein encoded by this gene binds nucleosomal DNA and is associated with transcriptionally active chromatin. Along with a similar protein, HMG17, the encoded protein may help maintain an open chromatin configuration around transcribable genes. [provided by RefSeq, Aug 2011]
10	UROS	uroporphyrinogen III synthase	The protein encoded by this gene catalyzes the fourth step of porphyrin biosynthesis in the heme biosynthetic pathway. Defects in this gene cause congenital erythropoietic porphyria (Gunther's disease). [provided by RefSeq, Jul 2008]
16	ANKRD11	ankyrin repeat domain 11	This locus encodes an ankryin repeat domain-containing protein. The encoded protein inhibits ligand-dependent activation of transcription. Mutations in this gene have been associated with KBG syndrome, which is characterized by macrodontia, distinctive craniofacial features, short stature, skeletal anomalies, global developmental delay, seizures and intellectual disability. Alternatively spliced transcript variants have been described. Related pseudogenes exist on chromosomes 2 and X. [provided by RefSeq, Jan 2012]

Supplemental Table S3 Continued. Isocyanate biomarker-associated CpG genes that have descriptions on NCBI.

3B) HDA levels in urine

CHR	Gene	Full Gene Name	Gene Function
16	ZFHX3	zinc finger homeobox 3	This gene encodes a transcription factor with multiple homeodomains and zinc finger motifs, and regulates myogenic and neuronal differentiation. The encoded protein suppresses expression of the alpha-fetoprotein gene by binding to an AT-rich enhancer motif. The protein has also been shown to negatively regulate c-Myb, and transactivate the cell cycle inhibitor cyclin-dependent kinase inhibitor 1A (also known as p21CIP1). This gene is reported to function as a tumor suppressor in several cancers, and sequence variants of this gene are also associated with atrial fibrillation. Multiple transcript variants expressed from alternate promoters and encoding different isoforms have been found for this gene. [provided by RefSeq, Sep 2009]
7	FZD9	frizzled class receptor 9	Members of the 'frizzled' gene family encode 7-transmembrane domain proteins that are receptors for Wnt signaling proteins. The FZD9 gene is located within the Williams syndrome common deletion region of chromosome 7, and heterozygous deletion of the FZD9 gene may contribute to the Williams syndrome phenotype. FZD9 is expressed predominantly in brain, testis, eye, skeletal muscle, and kidney. [provided by RefSeq, Jul 2008]
19	UNC13A	unc-13 homolog A	This gene encodes a member of the UNC13 family. UNC13 proteins bind to phorbol esters and diacylglycerol (DAG) and play important roles in neurotransmitter release at synapses. Single nucleotide polymorphisms in this gene may be associated with sporadic amyotrophic lateral sclerosis. [provided by RefSeq, Feb 2012]
19	FUZ	fuzzy planar cell polarity protein	This gene encodes a planar cell polarity protein that is involved in ciliogenesis and directional cell movement. Knockout studies in mice exhibit neural tube defects and defective cilia, and mutations in this gene are associated with neural tube defects in humans. Alternatively spliced transcript variants have been found for this gene. [provided by RefSeq, Jul 2012]
19	MED25	mediator complex subunit 25	This gene encodes a component of the transcriptional coactivator complex termed the Mediator complex. This complex is required for transcription of most RNA polymerase II-dependent genes. The encoded protein plays a role in chromatin modification and in preinitiation complex assembly. Mutations in this gene are associated with Charcot-Marie-Tooth disease type 2B2. [provided by RefSeq, Apr 2010]

Supplemental Table S3 Continued. Isocyanate biomarker-associated CpG genes that have descriptions on NCBI.

3C) TAHI levels in plasma

CHR	Gene	Full Gene Name	Gene Function
11	SNX19	sorting nexin 19	Islet antigen-2 (IA-2) is an autoantigen in type 1 diabetes and plays a role in insulin secretion. IA-2 is found in dense-core secretory vesicles and interacts with the product of this gene, a sorting nexin. In mouse pancreatic beta-cells, the encoded protein influenced insulin secretion by stabilizing the number of dense-core secretory vesicles. [provided by RefSeq, Dec 2016]
12	KRT6A	keratin 6A	The protein encoded by this gene is a member of the keratin gene family. The type II cytokeratins consist of basic or neutral proteins which are arranged in pairs of heterotypic keratin chains coexpressed during differentiation of simple and stratified epithelial tissues. As many as six of this type II cytokeratin (KRT6) have been identified; the multiplicity of the genes is attributed to successive gene duplication events. The genes are expressed with family members KRT16 and/or KRT17 in the filiform papillae of the tongue, the stratified epithelial lining of oral mucosa and esophagus, the outer root sheath of hair follicles, and the glandular epithelia. This KRT6 gene in particular encodes the most abundant isoform. Mutations in these genes have been associated with pachyonychia congenita. In addition, peptides from the C-terminal region of the protein have antimicrobial activity against bacterial pathogens. The type II cytokeratins are clustered in a region of chromosome 12q12-q13. [provided by RefSeq, Oct 2014]
4	MAPK10	mitogen-activated protein kinase 10	The protein encoded by this gene is a member of the MAP kinase family. MAP kinases act as integration points for multiple biochemical signals, and thus are involved in a wide variety of cellular processes, such as proliferation, differentiation, transcription regulation and development. This kinase is specifically expressed in a subset of neurons in the nervous system, and is activated by threonine and tyrosine phosphorylation. Targeted deletion of this gene in mice suggests that it may have a role in stress-induced neuronal apoptosis. Alternatively spliced transcript variants encoding different isoforms have been described for this gene. A recent study provided evidence for translational readthrough in this gene, and expression of an additional C-terminally extended isoform via the use of an alternative in-frame translation termination codon. [provided by RefSeq, Dec 2017]
1	LEPR	leptin receptor	The protein encoded by this gene belongs to the gp130 family of cytokine receptors that are known to stimulate gene transcription via activation of cytosolic STAT proteins. This protein is a receptor for leptin (an adipocyte-specific hormone that regulates body weight), and is involved in the regulation of fat metabolism, as well as in a novel hematopoietic pathway that is required for normal lymphopoiesis. Mutations in this gene have been associated with obesity and pituitary dysfunction. Alternatively spliced transcript variants encoding different isoforms have been described for this gene. It is noteworthy that this gene and LEPROT gene (GeneID:54741) share the same promoter and the first 2 exons, however, encode distinct proteins (PMID:9207021). [provided by RefSeq, Nov 2010]

Supplemental Table S3 Continued. Isocyanate biomarker-associated CpG genes that have descriptions on NCBI.

3D) TAHI levels in urine

CHR	Gene	Full Gene Name	Gene Function
9	COL27A1	collagen type XXVII alpha 1 chain	This gene encodes a member of the fibrillar collagen family, and plays a role during the calcification of cartilage and the transition of cartilage to bone. The encoded protein product is a preproprotein. It includes an N-terminal signal peptide, which is followed by an N-terminal propetide, mature peptide and a C-terminal propeptide. The N-terminal propeptide contains thrombospondin N-terminal-like and laminin G-like domains. The mature peptide is a major triple-helical region. The C-terminal propeptide, also known as COLFI domain, plays crucial roles in tissue growth and repair. Mutations in this gene cause Steel syndrome. Alternatively spliced transcript variants have been found, but the full-length nature of some variants has not been determined. [provided by RefSeq, Sep 2014]
8	CHD7	chromodomain helicase DNA binding protein 7	This gene encodes a protein that contains several helicase family domains. Mutations in this gene have been found in some patients with the CHARGE syndrome. Two transcript variants encoding different isoforms have been found for this gene. [provided by RefSeq, Oct 2015]
19	NOSIP	nitric oxide synthase interacting protein	The protein encoded by this gene may modulate the activity and localization of nitric oxide synthase (endothelial and neuronal) and thus nitric oxide production. Alternative splicing results in multiple transcript variants that encode the same protein. [provided by RefSeq, Aug 2012]
16	ALG1	ALG1 chitobiosyldiphosphodolichol beta-mannosyltransferase	The enzyme encoded by this gene catalyzes the first mannosylation step in the biosynthesis of lipid-linked oligosaccharides. This gene is mutated in congenital disorder of glycosylation type Ik. [provided by RefSeq, Dec 2008]
16	C16orf89	chromosome 16 open reading frame 89	This gene is expressed predominantly in the thyroid. Based on expression patterns similar to thyroid transcription factors and proteins, this gene may function in the development and function of the thyroid. Multiple transcript variants encoding different isoforms have been found for this gene. [provided by RefSeq, Oct 2011]

1A) HDA Plasma Levels

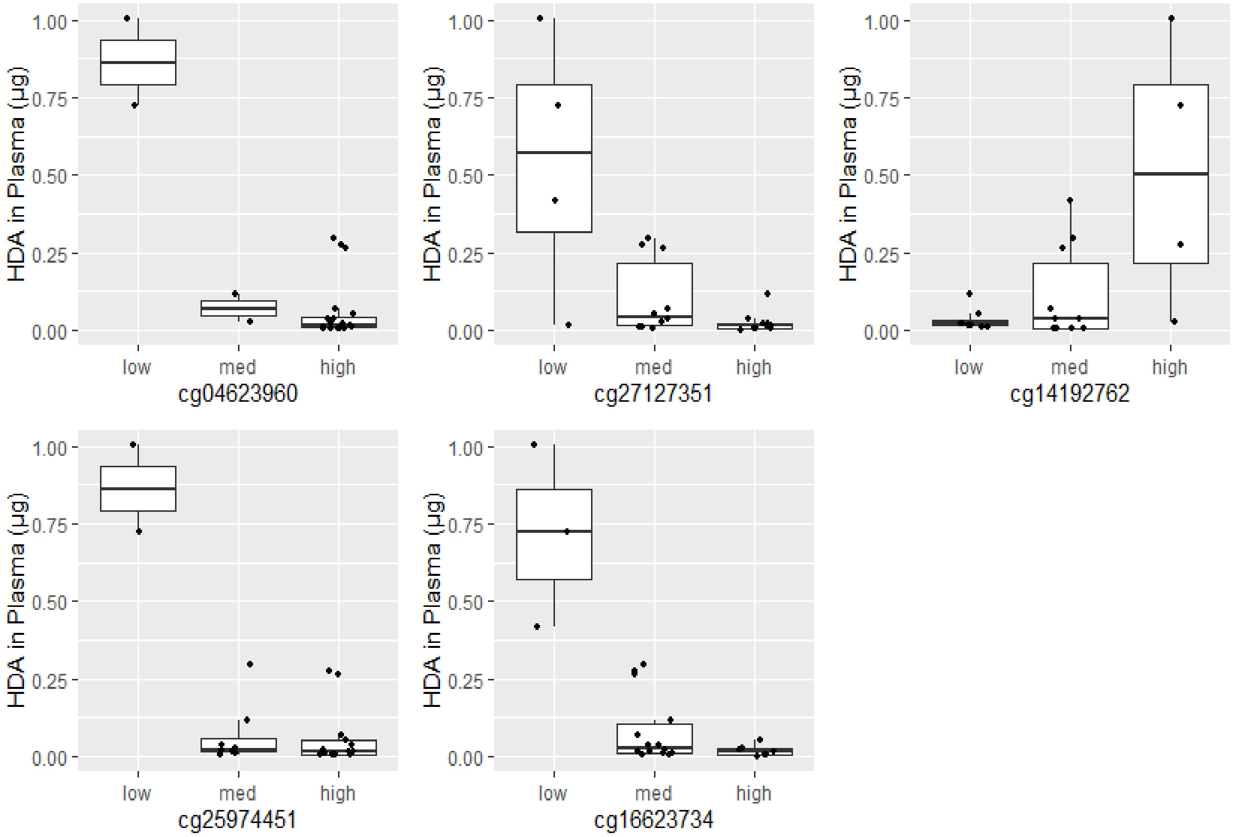

CpG	Methylation Level Bin Ranges		
	Low	Medium (med)	High
cg04623960	[0.904, 0.919]	(0.919, 0.934]	(0.934, 0.949]
cg27127351	[0.978, 0.98]	(0.98, 0.982]	(0.982, 0.984]
cg14192762	[0.00785, 0.0105]	(0.0105, 0.0132]	(0.0132, 0.0158]
cg25974451	[0.921, 0.934]	(0.934, 0.947]	(0.947, 0.96]
cg16623734	[0.934, 0.945]	(0.945, 0.956]	(0.956, 0.967]

Supplemental Figure S1. Boxplots showing the CpG methylation levels (binned into three equal groups for each CpG's values as shown in the table) versus isocyanate biomarker levels for HDA levels in plasma (1A), HDA levels in urine (1B), TAHI levels in plasma (1C), and TAHI levels in urine (1D). The binning of methylation was performed after analysis to make the figures more comparable to the GWAS boxplots we published previously (Taylor et al, 2020: https://doi.org/10.3389/fgene.2020.00836).

1B) HDA Urine Levels

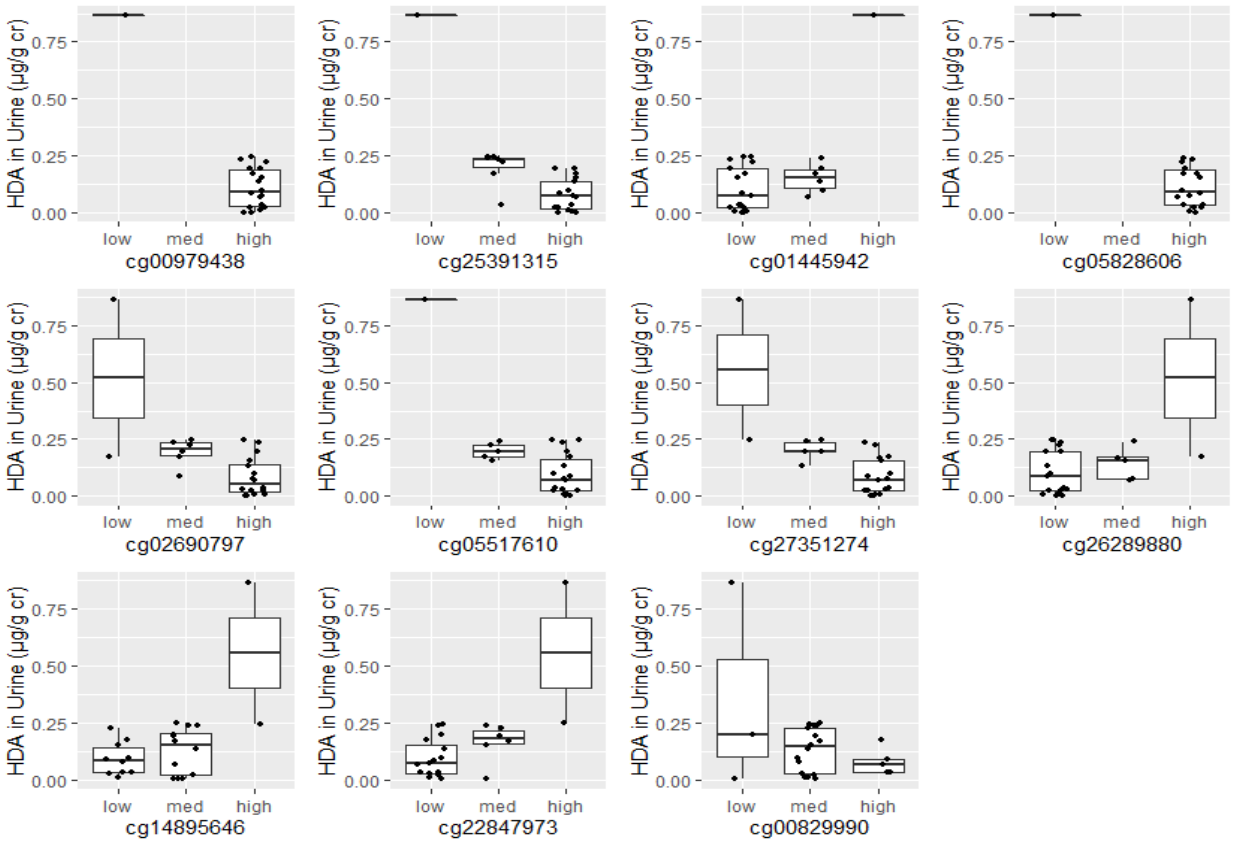

CpG	Methylation Level Bin Ranges		
	Low	Medium (med)	High
cg00979438	[0.665, 0.769]	–	(0.873, 0.978]
cg25391315	[0.885, 0.912]	(0.912, 0.939]	(0.939, 0.966]
cg01445942	[0.0259, 0.0322]	(0.0322, 0.0385]	(0.0385, 0.0448]
cg05828606	[0.684, 0.78]	–	(0.875, 0.971]
cg02690797	[0.731, 0.781]	(0.781, 0.831]	(0.831, 0.881]
cg05517610	[0.501, 0.614]	(0.614, 0.728]	(0.728, 0.841]
cg27351274	[0.862, 0.894]	(0.894, 0.926]	(0.926, 0.958]
cg26289880	[0.00772, 0.0118]	(0.0118, 0.0158]	(0.0158, 0.0198]
cg14895646	[0.0177, 0.0211]	(0.0143, 0.0177]	(0.0211, 0.0244]
cg22847973	[0.067, 0.143]	(0.143, 0.22]	(0.22, 0.296]
cg00829990	[0.0972, 0.173]	(0.173, 0.249]	(0.249, 0.325]

Supplemental Figure S1 Continued. Boxplots showing the CpG methylation levels (binned into three equal groups for each CpG's values as shown in the table) versus isocyanate biomarker levels for HDA levels in plasma (1A), HDA levels in urine (1B), TAHI levels in plasma (1C), and TAHI levels in urine (1D). The binning of methylation was performed after analysis to make the figures more comparable to the GWAS boxplots we published previously (Taylor et al, 2020: https://doi.org/10.3389/fgene.2020.00836).

1C) TAHI Plasma Levels

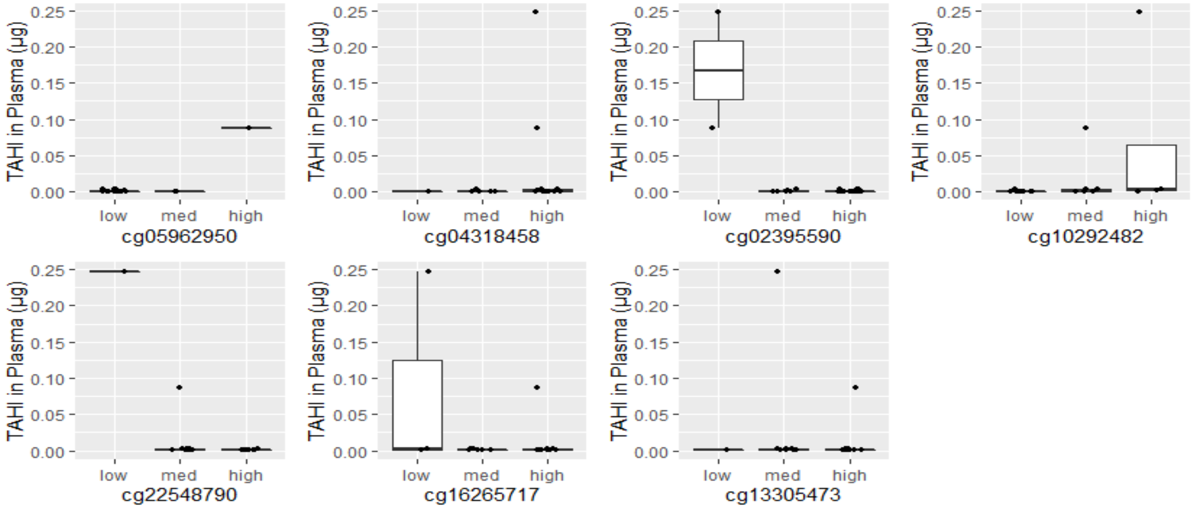

CpG	Methylation Level Bin Ranges		
	Low	Medium (med)	High
cg05962950	[0.0099, 0.0259]	(0.0259, 0.0418]	(0.0418, 0.0578]
cg04318458	[0.924, 0.936]	(0.936, 0.949]	(0.949, 0.961]
cg02395590	[0.83, 0.866]	(0.866, 0.902]	(0.902, 0.937]
cg10292482	[0.00866, 0.012]	(0.012, 0.0154]	(0.0154, 0.0188]
cg22548790	[0.951, 0.957]	(0.957, 0.964]	(0.964, 0.971]
cg16265717	[0.946, 0.955]	(0.955, 0.964]	(0.964, 0.972]
cg13305473	[0.86, 0.881]	(0.881, 0.902]	(0.902, 0.924]

Supplemental Figure S1 Continued. Boxplots showing the CpG methylation levels (binned into three equal groups for each CpG's values as shown in the table) versus isocyanate biomarker levels for HDA levels in plasma (1A), HDA levels in urine (1B), TAHI levels in plasma (1C), and TAHI levels in urine (1D). The binning of methylation was performed after analysis to make the figures more comparable to the GWAS boxplots we published previously (Taylor et al, 2020: https://doi.org/10.3389/fgene.2020.00836).

1D) TAHI Urine Levels

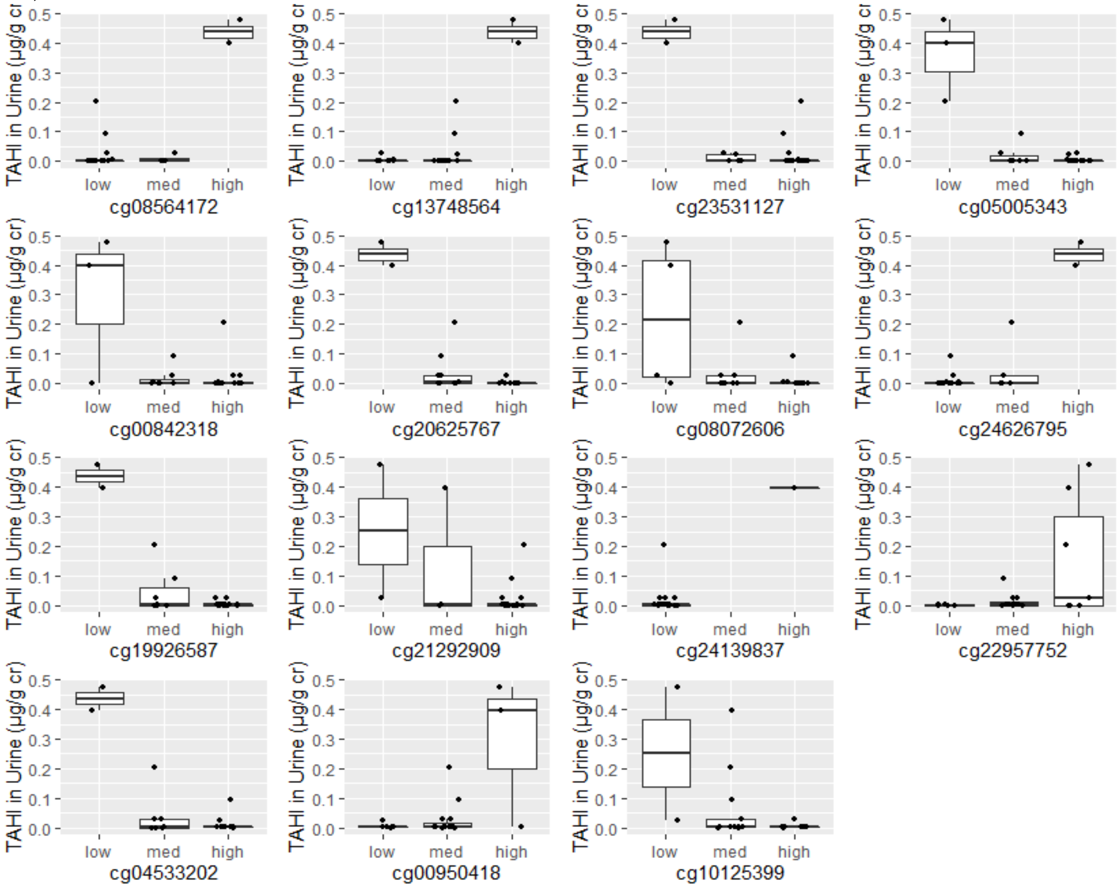

CpG	Methylation Level Bin Ranges		
	Low	Medium (med)	High
cg08564172	[0.0263, 0.0476]	(0.0476, 0.0688]	(0.0688, 0.0901]
cg13748564	[0.011, 0.0258]	(0.0258, 0.0407]	(0.0407, 0.0555]
cg23531127	[0.927, 0.945]	(0.945, 0.962]	(0.962, 0.98]
cg05005343	[0.896, 0.917]	(0.917, 0.937]	(0.937, 0.958]
cg00842318	[0.86, 0.884]	(0.884, 0.908]	(0.908, 0.932]
cg20625767	[0.813, 0.843]	(0.843, 0.873]	(0.873, 0.903]
cg08072606	[0.93, 0.937]	(0.937, 0.944]	(0.944, 0.952]
cg24626795	[0.00943, 0.0178]	(0.0178, 0.0262]	(0.0262, 0.0346]
cg19926587	[0.863, 0.887]	(0.887, 0.912]	(0.912, 0.937]
cg21292909	[0.884, 0.912]	(0.912, 0.941]	(0.941, 0.969]
cg24139837	[0.00842, 0.0589]	–	(0.109, 0.16]
cg22957752	[0.523, 0.546]	(0.546, 0.569]	(0.569, 0.592]
cg04533202	[0.757, 0.803]	(0.803, 0.848]	(0.848, 0.893]
cg00950418	[0.00497, 0.00969]	(0.00969, 0.0144]	(0.0144, 0.0191]
cg10125399	[0.458, 0.604]	(0.604, 0.75]	(0.75, 0.896]

Supplemental Figure S1 Continued. Boxplots showing the CpG methylation levels (binned into three equal groups for each CpG's values as shown in the table) versus isocyanate biomarker levels for HDA levels in plasma (1A), HDA levels in urine (1B), TAHI levels in plasma (1C), and TAHI levels in urine (1D). The binning of methylation was performed after analysis to make the figures more comparable to the GWAS boxplots we published previously (Taylor et al, 2020: https://doi.org/10.3389/fgene.2020.00836).
